# Hyperpure chlorine dioxide versus chlorhexidine in intra-oral halitosis (ODOR trial) – protocol of a double-blinded, double-arm, parallel non-inferiority pilot randomized controlled trial

**DOI:** 10.1038/s41405-024-00221-8

**Published:** 2024-05-20

**Authors:** Eszter Ágnes Szalai, Brigitta Teutsch, Viktória Babay, Adél Galvács, Péter Hegyi, Péter Hársfalvi, Róbert Pál, Gábor Varga, Zsolt M. Lohinai, Beáta Kerémi

**Affiliations:** 1 Department of Restorative Dentistry and Endodontics, Semmelweis University, Budapest, Hungary; 2 Centre for Translational Medicine, Semmelweis University, Budapest, Hungary; 3 Department of Radiology, Medical Imaging Centre, Semmelweis University, Budapest, Hungary; 4 Institute for Translational Medicine, Medical School, University of Pécs, Pécs, Hungary; 5 Faculty of Dentistry, Semmelweis University, Budapest, Hungary; 6 Division of Pancreatic Diseases, Semmelweis University, Budapest, Hungary; 7 University of Veterinary Medicine Budapest, Department of Biostatistics, Budapest, Hungary; 8 BiTrial Clinical Research, Budapest, Hungary; 9 Today Science Ltd., Diósd, Hungary; 10 Department of Oral Biology, Semmelweis University, Budapest, Hungary

**Keywords:** Oral diseases, Halitosis

## Abstract

**Introduction:**

Intra-oral halitosis (IOH) is the most common type of bad breath; its consequences impair quality of life. However, evidence-based treatment protocols and guidelines are lacking. Our aim is to investigate the effectiveness of chlorine dioxide as an applicable complementary treatment modality in IOH after tongue cleaning.

**Methods and analysis:**

The ODOR trial will be a single-center, double-blinded, parallel-group, double-armed pilot randomized controlled trial with a non-inferiority design. The efficacy of hyperpure chlorine dioxide will be compared to chlorhexidine mouthwash. We plan to investigate the short-term effects of the intervention over a 3-h period. The primary endpoint will be changes in organoleptic test scores. At the end of the pilot investigation of the first 30 patients each, sample size calculation will be performed. If feasible, the investigators will continue the study by enrolling more patients.

**Trial registration:**

The trial has been registered at ClinicalTrials.gov (NCT06219226).

## Introduction

### Background

The prevalence of halitosis is approximately 31.8% and is constantly increasing [1]. Intra-oral halitosis (IOH) is the most common type of bad breath [2]. The main consequences of halitosis are feelings of inadequacy, depression, anxiety, sensitivity, anger, stress [3], and impaired oral health-related quality of life (OHRQoL) [4].

Mouthwashes are the subsequent treatment of IOH after toothbrushing and tongue cleaning [5–8]; however, guidelines for the management of IOH still do not exist. Several types of mouthwashes are available on the market. Although most of the currently used ones, such as those containing chlorhexidine or alcohol, are effective; however, they are not recommended for daily use due to their potential side effects [9] such as tooth discoloration [10], and false taste sensations [11]. Chlorhexidine is considered to be the gold standard mouthwash for its potent antimicrobial properties and effectiveness in reducing plaque and gingivitis [12]. However, when it comes to intra-oral halitosis, the evidence supporting the efficacy of chlorhexidine is still uncertain [13].

Mouthwashes containing chlorine dioxide (ClO_2_) have a doubled effect of reducing IOH; they reduce the amount of bacteria together with the volatile sulfur compounds [14–16], which are the main components of IOH. Their main advantage is the lack of known side effects and efficacy in low concentrations [17]. Most ClO_2_ mouthwashes contain a stabilized form of ClO_2_ [18–21]. However, they are assumed to be less effective than hyperpure ClO_2_ due to lower concentrations of active ClO_2_ molecules and contamination with other ingredients [14, 22]. In an in vitro study, the hyperpure ClO_2_ solution showed superior effectiveness to chlorhexidine against aerobic bacteria and Candida; furthermore, its biofilm-dissolving effect is significantly higher [23].

Several studies have already investigated stabilized ClO_2_ mouthwashes in IOH [20, 24–27]; a meta-analysis [28] found them effective in halitosis compared to placebo. However, none of the previous studies have investigated the efficacy of a hyperpure version of ClO_2_ in IOH compared to chlorhexidine, which indicates the relevance of this randomized controlled trial (RCT).

### Objectives

We aim to investigate the effectiveness of hyperpure ClO_2_ in IOH with organoleptic measurement as the primary endpoint.

We hypothesize that hyperpure ClO_2_-containing mouthwash is non-inferior to gold standard chlorhexidine mouthwash in IOH, reducing the halitosis level measured by the organoleptic test score.

### Trial design

This study protocol is designed as a pilot, single-center, double-blinded, parallel-group, double-armed RCT with a non-inferiority design. We plan to investigate the short-term effects over a 3-h period.

## Methods

### Study protocol development

The SC and ITAB members developed the first version of the trial protocol following the Standard Protocol Items Recommendations for Interventional Trials (SPIRIT) 2013 Statement [29].

### Study setting

The study will be performed with one urban center at the Department of Restorative Dentistry and Endodontics, Semmelweis University (Budapest, Hungary).

### Eligibility criteria

#### Inclusion criteria

- ►Aged 18 or older
- ►Organoleptic test score (OLS) ≥ 2 for IOH (A trained dentist will perform the measurements.)
- ►Patients with at least 20 teeth.
- ►Eight hours of use of scented oral hygiene product, 4 h of eating, and 2 h of drinking restriction
- ►Restriction of alcohol, caffeine, perfume usage, and food intake with characteristic smell on the day of investigation

#### Exclusion criteria

- ►Medical history of systemic and infectious diseases (e.g., hepatitis, HIV, tuberculosis)
- ►Antibiotic use within a month before or during the study or any regular medication
- ►Extraoral halitosis (distinguished by observing the nasal breath)
- ►Eat foods linked to oral malodor (e.g., garlic) on the day before and on the day of sampling, as well as wear heavily fragrant cosmetics on that day
- ►Patients with removable dentures
- ►Smokers (cigars, cigarettes, pipes, chewing tobacco, e-cigarette, or vaping products used in the last month)

### Interventions

The two arms will be the following:

1. Hyperpure ClO_2_ mouthwash (Solumium Coral®)
2. Chlorhexidine mouthwash (Curasept ADS 220®)

Enrollment begins January 18, 2024.

We will perform randomization if eligibility criteria are fulfilled. After baseline data have been recorded, interventions will be performed.The test group will use a 25 ml 10-fold dilution of hyperpure 0.03% ClO_2_ (Solumium Coral®, final concentration: 0.003%) [30], and the control group will use chlorhexidine-containing mouthwashes (Curasept ADS 220, 0.2%). Rinsing will last 2 × 15 s in both groups as recommended by the manufacturer (2 × 12.5 ml) [31]. Intervention and control mouthwashes will be portioned into equally dark glasses.

### Outcomes

#### Primary outcome

The primary endpoint of the study will be the changes in organoleptic test scores as measured by a trained dentist with the gold standard 6-point (0–5) intensity scale [32].

Tests will be performed three times (Table 1): at baseline, immediately after and 3 h after rinsing with mouthwash. Two changes will be measured between the following time points: 1) baseline - immediately after rinsing, and 2) baseline - 3 h later; as suggested by Yaegaki et al. in their halitosis research [33].

**Table 1:** Participant timeline.

			Study period			
	Enrollment	Allocation	Post allocation			Close-out
Time point	Starting from January 18, 2024	Baseline	Intervention	Immediately after mouthwash use	3 h later	December 1, 2025
Enrollment:						
Eligibility screen	X					
Informed consent		X				
Randomization		X				
Interventions:						
Hyperpure chlorine dioxide			X			
Chlorhexidine			X			
Assessments:						
Organoleptic score	X	X		X	X	
Volatile sulfur compound measurement with a gas chromatograph		X		X	X	
Self-perceived halitosis		X		X	X	
Subjective experiences, adverse events				X	X	
Statistical analysis						X

Schedule of enrollment, interventions, and assessments.

#### Secondary outcomes

1. We plan to measure the changes in volatile sulfur compounds, such as hydrogen sulfide, methyl mercaptan, and dimethyl sulfide with gas chromatography-mass spectrometry (GC/MS) at the same time points as the primary endpoint. It is an instrumental technique comprising a gas chromatograph coupled to a mass spectrometer, which allows complex mixtures of chemicals to be separated, identified and quantified. Calibration will be performed with a gas mixture produced by Linde (100 ppb H_2_S, 500 ppb CH_3_SH, 1000 ppb (CH_3_)_2_S). Samples will be collected in Teflon-coated bags and delivered to the measurement site immediately after sampling.
2. Self-perceived halitosis. We will collect these data on a visual analog scale (Slider (RedCAP), visual analog scale coded as values 0–100) at the time mentioned above.
3. Side effects. (e.g., tooth discoloration, signs of allergic reactions, subjective experiences: unpleasant taste, false taste or burning sensation, pain, and changes in salivary flow).

#### Sample size

We plan to conduct a study with a sample exploratory nature because the specific intervention has not yet revealed data in IOH in the literature. In the pilot phase, 30 participants each will be investigated in two groups. Then, the results of the primary outcome are used for sample size estimation. If the pilot can be continued, we will use it as an interim analysis in our study.

#### Recruitment

The primary site of recruitment is the Education Center of the Faculty of Dentistry (Semmelweis University, Budapest, Hungary), which receives on average 10,179 patients per month. The trial will be advertised on posters and social media.

#### Assignment of ﻿interventions

##### Allocation & blinding

Eligible patients will be randomly allocated to the intervention and control groups by REDCap. Randomization sequences will be generated by using the big stick design [34]. Trial participants and outcome assessors will be blinded. In addition, the data analyst will have access to the anonymized datasheet, without being aware of the allocated intervention. Dark, uniformly packaged, sealed bottles of mouthwash labeled A and B will contain the assigned interventions, which will be given to patients after the randomization. A dental hygienist, who will not be involved in the assessments of the outcomes, will hand out the boxes with the assigned mouthwashes to the participants and supervise the rinsing process. The examiner will examine the patients in a separate room from rinsing without knowing their assigned intervention. The information in an opaque envelope about which mouthwashes are A and B will be kept in the safe of the guarantor of the trial.

#### Data collection, management, and analysis

##### Data collection methods and data management

The required data will be collected using prespecified electronic case report forms (REDCap) [35]. The principal investigator of the trial (ES) will anonymously handle and store participant data on a server for at least twenty years. Data collection forms can be found in Supplementary Documents 1.

Any data modifications will be recorded and checked by the statistician and the guarantor of the trial. Any missing or incorrect data will be reported by the statistician; and the data quality check module of REDCap will also be applied.

##### Statistical methods

We will report on baseline characteristics and differences between the two arms. For continuous primary and secondary endpoints, we will report the mean results of the groups and changes at the different time points. Due to the non-inferiority study design, we choose the non-inferiority limit (*d* = 1) as the most considerable clinically acceptable difference. For the side-effect questionnaire, we will create a summary table showing the frequency of adverse events and all patient comments. In the case of any missing data, sensitivity analyses will be conducted to determine the most suitable approach.

#### Monitoring

##### Data monitoring

The size and length of the trial make a data monitoring committee unnecessary. A statistician (PHá) and a clinical research specialist (BT), independent of the sponsor and the trial, will monitor data.

##### Safety and adverse events

Side effects of chlorine dioxide at this low concentration are not known yet [28]. Prolonged use of CHX-containing mouthwash can lead to several adverse events with a relatively high frequency and it is hypothesized that a specific type of control mouthwash may have fewer undesirable effects [12]. We will ask the patients about their experiences at each time point. If patients experience any adverse events, we will record these follow-up questionnaires. SC will verify the validity of adverse events; the relevant ones will be reported to the National Institute of Pharmacy and Nutrition (OGYÉI). If any safety concerns arise, we will stop the study immediately.

##### Withdrawal from study

Due to the length of the study, we expect a small number of eligible participants to withdraw or drop out. Previous studies [36–38] have reported a dropout rate of 10%.

##### Public and patient involvement

The protocol development phase involved three patients. We asked them to read the patient information leaflet and give informed consent after being introduced to the purpose, primary outcome, and protocol of the study. They thought that the written materials were transparent and informative. Our clinical question was deemed highly relevant by the patients. They said that the one-day caffeine restriction was too long, and that they could not imagine not brushing their teeth the night before the investigation. Thus, we reduced the time limits in the protocol, oral hygiene restriction from 12 to 8 h, and caffeine from one day to the day of the investigation. In addition, they expressed their preference for flavored mouthwashes.

#### Dissemination

##### Protocol amendments

Any potential deviation from the protocol will be discussed with the SC. If the team accepts the changes, they will be reported in the protocol registration and the final article with the data.

##### Publication policy

We plan to publish our results regardless of the results and the feasibility of the more extended version.

## Discussion

This research focuses on comparing the effectiveness of hyperpure ClO_2_ compared to chlorhexidine, as this specific intervention has not been thoroughly investigated in IOH. Currently, there is only one similar article [39] that compares chlorhexidine and ClO_2_. This particular intervention provides an opportunity to conduct a pilot trial. If our hypothesis is confirmed, we expect that hyperpure ClO_2_ is not inferior to chlorhexidine because of the dual action of ClO_2_. Moreover, a review [40] emphasized that it is a viable alternative to chlorhexidine. If the results are promising, further investigation of the interventions is needed for a more detailed and longer analysis. However, this detailed protocol will help improve studies in IOH research, and bring us one step closer to evidence-based treatment protocols.

## Strengths and limitations of the study

### Strengths

- The efficacy of hyperpure chlorine dioxide has not been investigated in the therapy of intra-oral halitosis yet.
- Gas chromatography calibrated with a standardized gas mixture will be used to assess halitosis.
- This study can be the basis for more extended studies.

### Limitations

- The long-term oral effects of hyperpure chlorine dioxide will not be monitored.
- First, 30 people each will be enrolled in two groups, then a sample size estimation will follow (lack of data on the intervention did not allow the sample size calculation). This will be a pilot study.

### Supplementary information

Supplementary Information

## Data Availability

The data supporting the findings of this study will be available upon reasonable request from the corresponding author, BK.
