# Supplementary material for: Hyperpure chlorine dioxide versus chlorhexidine in intra-oral halitosis (ODOR trial) – protocol of a double-blinded, double-arm, parallel non-inferiority pilot randomized controlled trial
Source: BDJ Open. 2024 May 20;10:35. doi: 10.1038/s41405-024-00221-8 (PMC11106080; doi:10.1038/s41405-024-00221-8)
Supplement: Supplementary file 1 — Supplementary Information [file 41405_2024_221_MOESM1_ESM.pdf]

Supplementary Documentum 1.: Data collection forms created with REDCap

Personal Data

Study ID

____

Consent Information

[Attachment: "ODOR_beleegyezo_nyilatkozat_18_felett_adatvedelem.pdf"]

Informed consent form

____

Date of subject signed consent

____
(YYYY-MM-DD)

Patient information statement

[Attachment: "ODOR_betegtajekoztato_18_felett_adatvedelem.pdf"]

Patient information statement

____

Date of subject signed patient information statement

____
(YYYY-MM-DD)

Patient personal details

Last Name

____

First Name

____

Insurance number

____
(123456789)

Phone number

____
(36001112233)

E-mail address

____

Date of birth

____

Age	______________________<br>(calculated)
Ethnicity	◯ Asian<br>◯ Caucasian (White)<br>◯ African (Black)<br>◯ Latin-American<br>◯ Arabian<br>◯ Indian<br>◯ Roma<br>◯ Other
Gender	◯ Female<br>◯ Male<br>◯ Other<br>◯ Prefer not to say

In the case of female patients

Is the patient pregnant or breastfeeding?	◯ Yes<br>◯ No
If the patient is female and not pregnant.<br>The date of the last period started	______________________
Name of the doctor providing information	______________________<br>(First name Last name)
Signature of the doctor providing information	___________________________

Powered by REDCap

Randomization

Is the participant eligible for the study based on inclusion and exclusion criteria?	◯ Yes<br>◯ No
Randomization	◯ Mouthwash A<br>◯ Mouthwash B
Participant randomized?	◯ Igen<br>◯ Nem
Date randomized:	________________________
If eligible and NOT randomized, indicate reason:	◯ Failed to return<br>◯ Declined participation<br>◯ Other
If Other, pleae specify:	______________________________

Powered by REDCap

Baseline Data

Date of baseline visit ______________________

Organoleptic test score (0-6) ______________________

Self-perceived halitosis

no bad breath — very severe bad breath

(Place a mark on the scale above)

Gas Chromatography

Time of the sample collection ______________________

Time of the measurement ______________________

Hydrogen sulfide (ng/10 mL) ______________________

Methyl mercaptan (ng/10 mL) ______________________

Dimethyl sulfide (ng/ 10mL) ______________________

5 minutes follow up

Organoleptic test score (0-6) __________________________________

Self-perceived halitosis

no bad breath — very severe bad breath

(Place a mark on the scale above)

Gas Chromatography

Time of the sample collection __________________________________

Time of the measurement __________________________________

Hydrogen sulfide (ng/10 mL) __________________________________

Methyl mercaptan (ng/10 mL) __________________________________

Dimethyl sulfide (ng/ 10mL) __________________________________

Mouthwashes' side effects or complication indicator

- ◯ Yes
- ◯ No
- ◯ Unknown

Type of side effects

- ☐ abnormal taste sensation
- ☐ burning sensation
- ☐ unpleasant taste
- ☐ teeth staining
- ☐ mucosal lesion
- ☐ pain
- ☐ changes in salivary flow
- ☐ signs of allergic reactions (itching or redness or rash or swelling of the lips, mouth or throat)
- ☐ other

Other side effects __________________________________

Side effects severity

◯ Grade 1 (mild) ◯ Grade 2 (moderate) ◯ Grade 3 (severe) ◯ Grade 4 (potentially life-threatening)

(GRADE 1 (mild) Transient (goes away after a short time) or mild discomfort. No limitation in activity. No medical intervention required. GRADE 2 (moderate) Your daily activity is affected mild to moderately. You need some assistance. No or only minimal medical intervention is needed. GRADE 3 (severe) Your daily activity is markedly reduced. Some assistance is usually needed. Medical intervention is needed. Hospitalisation or hospice care possible. GRADE 4 (potentially life threatening) Extreme limitation to daily activity. Significant assistance required. Significant medical intervention, hospital or hospice care very likely.)

Powered by REDCap

3 hours follow up

Organoleptic test score (0-6) ______________________

Self-perceived halitosis

no bad breath — very severe bad breath

(Place a mark on the scale above)

Gas Chromatography

Time of the sample collection ______________________

Time of the measurement ______________________

Hydrogen sulfide (ng/10 mL) ______________________

Methyl mercaptan (ng/10 mL) ______________________

Dimethyl sulfide (ng/ 10mL) ______________________

Mouthwashes' side effects or complication indicator

- ◯ Yes
- ◯ No
- ◯ Unknown

Type of side effects

- ☐ abnormal taste sensation
- ☐ burning sensation
- ☐ unpleasant taste
- ☐ teeth staining
- ☐ mucosal lesion
- ☐ pain
- ☐ changes in salivary flow
- ☐ signs of allergic reactions (itching or redness or rash or swelling of the lips, mouth or throat)
- ☐ other

Other side effects ______________________

Side effects severity

◯ Grade 1 (mild) ◯ Grade 2 (moderate) ◯ Grade 3 (severe) ◯ Grade 4 (potentially life-threatening)

Powered by REDCap

Completion Data

Study Completion Information

Has patient completed study?

◯ No
◯ Yes

Put a date if patient withdrew study

____

Reason patient withdrew from study

◯ Non-compliance
◯ Did not wish to continue in study
◯ Could not tolerate the supplement
◯ Hospitalization
◯ Other

General Comments

Comments

____

Powered by REDCap
